# Radium-223 as an Additional Therapeutic Strategy in Highly Selected Patients With Metastatic Breast Cancer: A Case Report

**DOI:** 10.3389/fonc.2022.896301

**Published:** 2022-05-27

**Authors:** Hélène Houssiau, Francois P. Duhoux, Didier François, Emmanuel Seront

**Affiliations:** ^1^ Department of Medical Oncology, Centre Hospitalier de Jolimont, Haine Saint Paul, Belgium; ^2^ Department of Medical Oncology, Cliniques Universitaires Saint Luc, Brussels, Belgium; ^3^ Department of Nuclear Medicine, Centre Hospitalier de Jolimont, Haine Saint Paul, Belgium

**Keywords:** breast cancer, radium-223, bone metastases, chemotherapy, positron emitted tomography

## Abstract

Radium-223 is commonly used in metastatic prostate cancer, targeting specifically bone metastases. The use of radium-223 remains, however, poorly evaluated in metastatic breast cancer. We report a case of radium-223 treatment in a 59-year-old patient with bone-only metastatic disease that progressed on multiple lines of systemic treatments. Radium-223 was very well tolerated and resulted in a regression of activity of bone metastases and in a 6-month progression-free survival. However, progression occurred in the liver, reflecting the fact that radium-223 should be combined with other systemic agents. This suggests that this therapeutic option is feasible and could be proposed in highly selected patients with bone metastatic disease outside of the prostate cancer field. Positron Emission Tomography appears also as a valuable tool for the evaluation of radium-223 efficacy.

## Introduction

Radium-223 is an alpha-emitting radiopharmaceutical that selectively targets bone metastases. The active fraction of radium-223 (radium-223 dichloride) is molecularly close to calcium and binds selectively to the bone by forming complexes with bone hydroxyapatite. By displaying avidity for areas affected by bone metastases, radium-233 emits alpha particles that cause DNA double-strand breaks in adjacent tumor cells, leading to a significant cytotoxic effect. The effect of radium-233 is relatively selective because its range of action is restricted to less than 100 μm, reducing damage to surrounding healthy tissue (1).

Radium-223 was approved by the Food and Drug Administration (FDA) in 2013 to treat patients with castration-resistant prostate cancer (CRPC) with bone metastases after failure of hormone therapy and chemotherapy (2). This approval was based on the phase III ALSYMPCA trial that randomized 921 CRPC patients with bone-only metastases in two groups: radium-223 (50kBq/kg every 4 weeks for six cycles) or placebo. Radium-223 resulted in a 3.6-month benefit in median overall survival compared to placebo (HR 0.70; 95% confidence interval (CI) 0.58-0.83, *P* = 0.0001) (2). Radium-223 treatment was well tolerated, with, as main toxicity, grade 1-2 digestive adverse events and reversible myelosuppression (2–4).

Results reporting the efficacy of radium-223 in monotherapy are limited in metastatic breast cancer; this is related to the difficulty to select patients with bone-only disease due to the high probability of breast cancer cells metastasizing in the extra-skeletal compartment and the challenge of monitoring correctly response to radium-223 (5–7). This case report describes the safety and the bone metastases control after five cycles of radium-223 in a heavily pretreated patient with breast cancer as well as the feasibility of subsequent platinum-based chemotherapy; the efficacy of radium-223 on bone metastases was described *via* different imaging tools.

## Case Description

**Figure 1:**
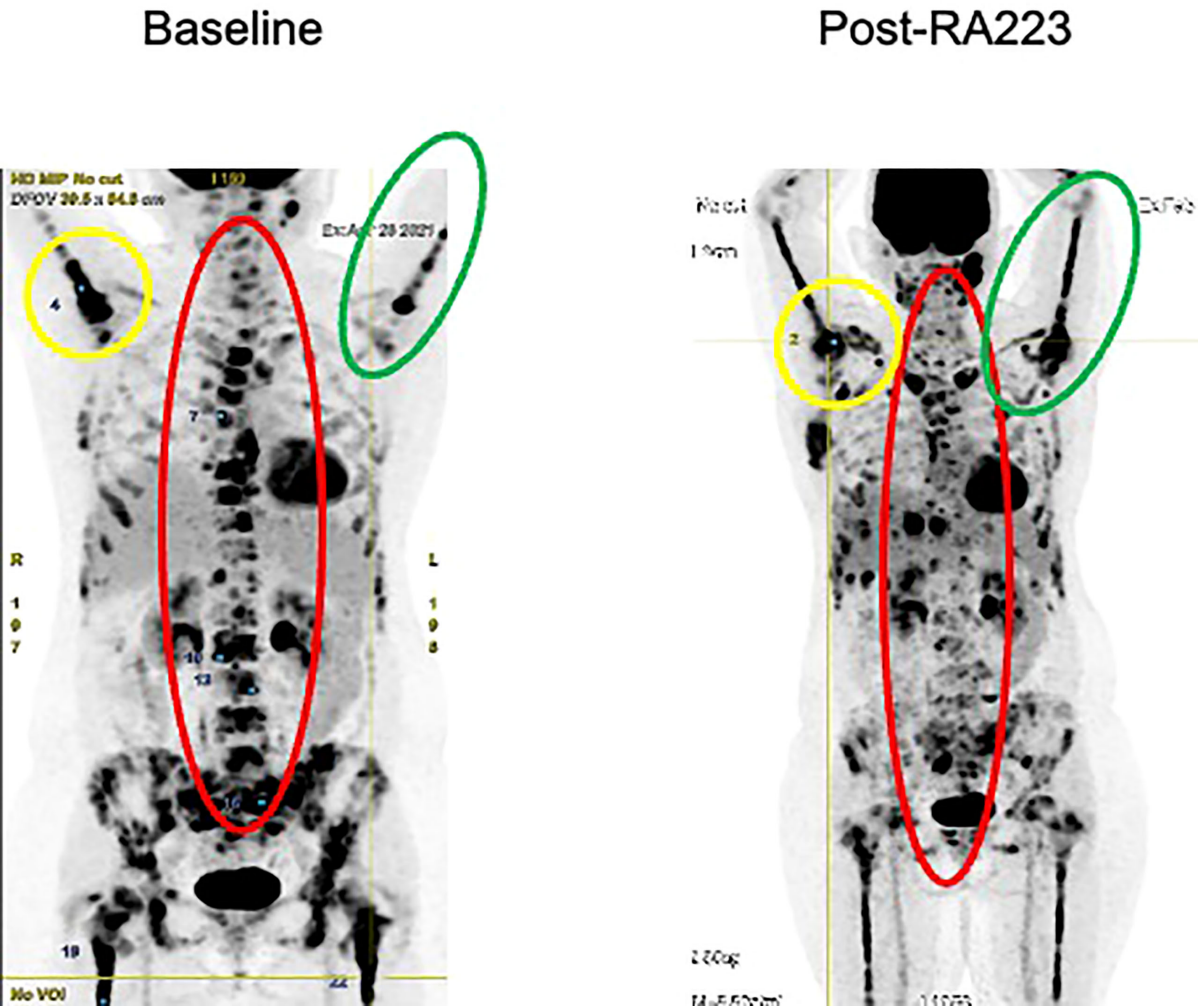
FDG PET-Scan before and after radium-223 treatment. The images circled in red depict a decrease of FDG uptake in axial skeletal metastases, reflecting a response to treatment. The images circled in green illustrate medullary regeneration in humerus and femur after treatment by showing homogeneization of FDG uptake. The images circled in yellow illustrate a decrease of FDG uptake in the right humeral head. For example, the Maximum Standardized Uptake Value (SUVmax) decreased of 59.2% in the at the 10th dorsal vertebra (12.38 in pre- to 5.05 in post- radium-223) and of 63.6% in the sacrum (14.91 in pre- to 5.43 in post-radium-223). The SUVmax of the Aorta bloodpool was 2.76 in pre- and 2.25 in post-radium-223 (-18%). The SUVmax in normal liver (reference) was 2.14 in pre- and 2.20 in post-radium-223 (+2.8%).

**Figure 2:**
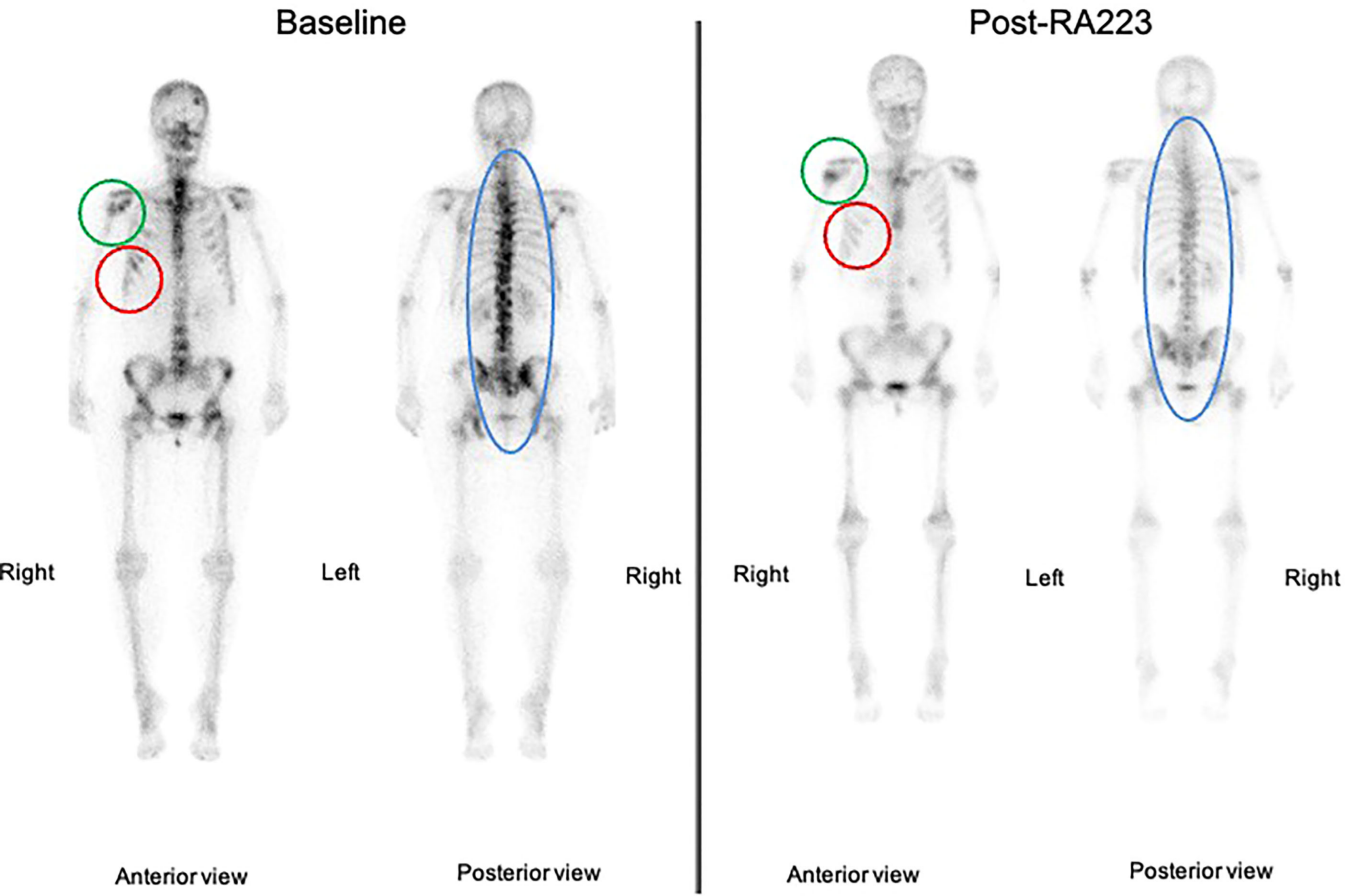
^99^mTc-HDP bone scan before and after radium-223 treatment. The images circled in red depict a decrease of ^99^Tc uptake reflecting decreased metastatic activity. The images circled in green show an increase in bone uptake in the right humeral head reflecting bone reconstruction (given a decrease of FDG uptake on PET-CT). The images circled in blue illustrate a global decrease in ^99^Tc uptake of the axial skeleton.

**Figure 3:**
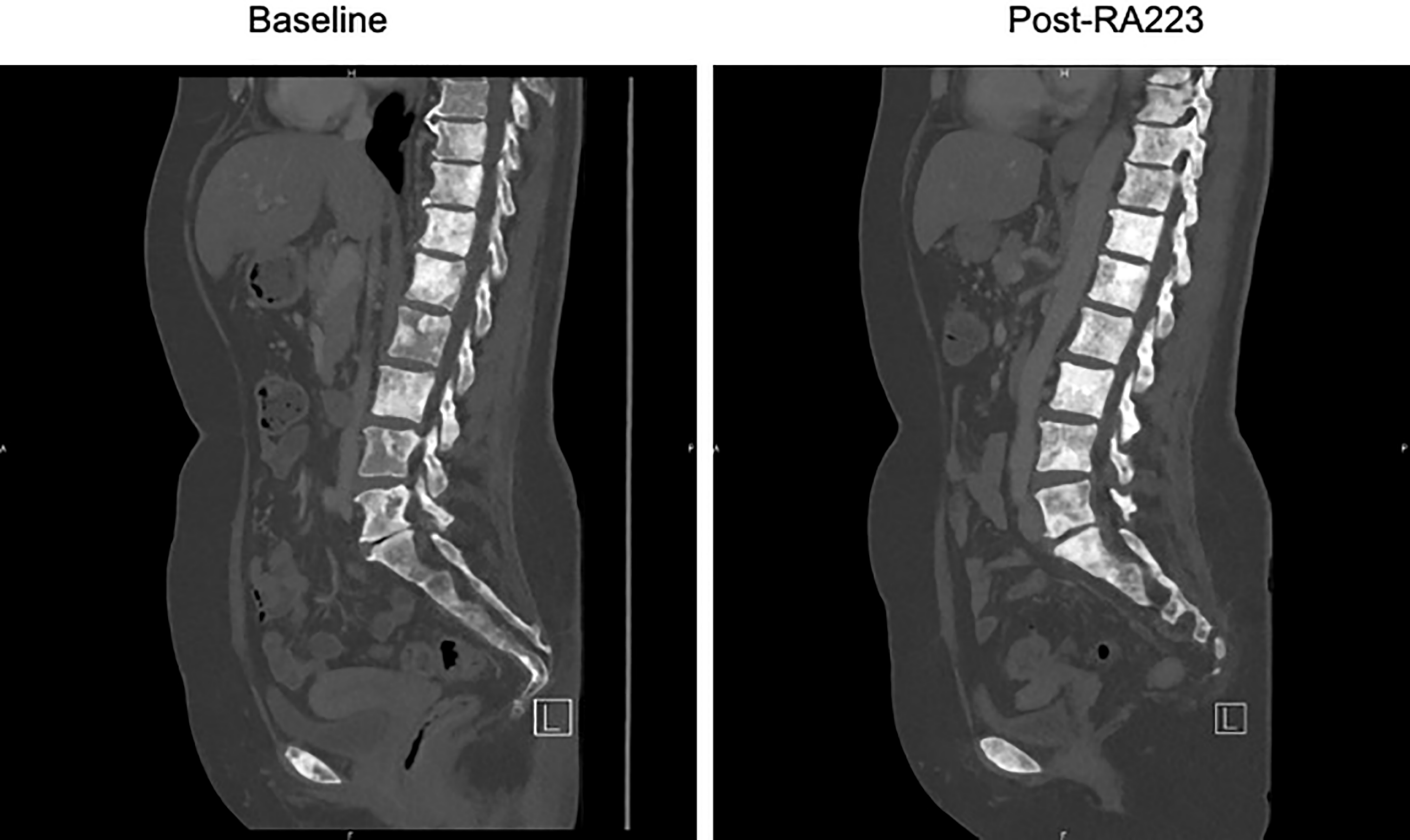
Lumbar spine CT-scan before and after Radium-223 treatment. The CT-scan performed after Radium-223 shows bone formation in lumbar vertebras.

**Figure 4:**
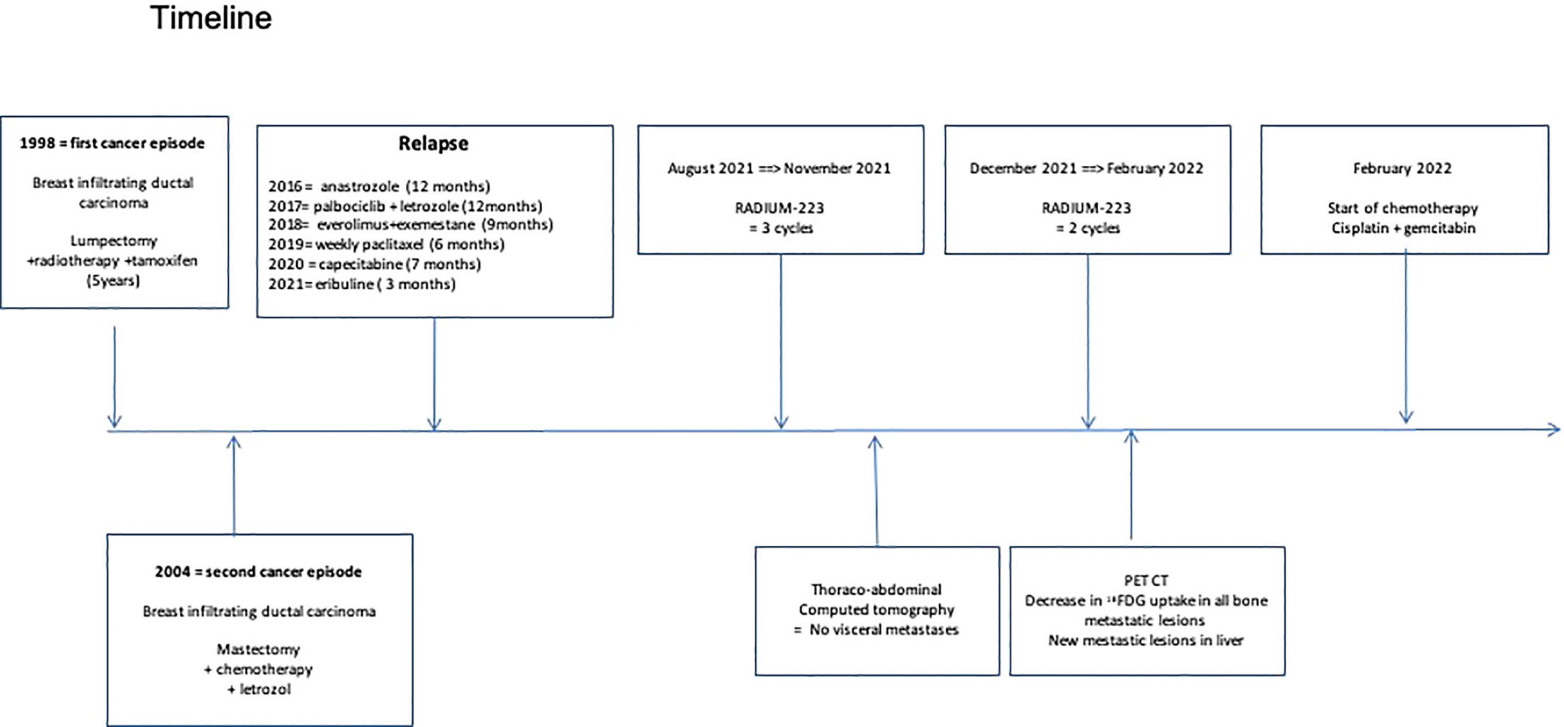
Timeline.

We report radium-223 treatment in a 59-year-old woman patient with metastatic breast cancer who progressed on multiple systemic cytotoxic treatments. This patient was first diagnosed with infiltrating ductal carcinoma of the right breast in 1998, treated with lumpectomy, radiotherapy, and 5 years of adjuvant tamoxifen. In 2004, a second homolateral breast cancer (infiltrating ductal carcinoma, grade 2, Estrogen Receptor (ER) 8/8, Progesterone Receptor (PgR) 2/8, HER2 0, Ki67 40%) was treated with mastectomy (as per the patient’s wish), chemotherapy (six cycles of 5-Fluorouracyl, Epirubicine, Cyclophosphamide) and 5 years of adjuvant letrozole. In 2016, an increase in CA15.3 level (62kU/L; normal range 15-30kU/L) led to the performance of a ^18^Fluorodeoxyglucose (FDG)-Positron Emission Tomography Computed tomography (PET-CT) that detected an axillary adenopathy and multiple bone metastases. Lymph node biopsy identified a grade 2 infiltrating ductal carcinoma (ER 8/8, PgR 8/8, HER2 0, Ki67 26%) and anastrozole therapy led to a rapid decrease in CA15.3 levels (30kU/L), the disappearance of the axillary adenopathy and decrease in FDG metabolism of bone lesions. Denosumab was started on a monthly basis administrated (subcutaneously) in association with calcium and D-vitamin. Twelve months later, in 2017, based on a rise of CA15.3 (120kU/L) and an increase in FDG metabolic uptake of bone lesions on PET-CT, anastrozole was switched to letrozole in combination with the cyclin-dependent kinase (CDK) 4/6 inhibitor palbociclib, which resulted in stability in CA15.3 and bone metastases metabolism for 10 months. In 2018, the increase in serum biomarkers (CA15.3 180kU/L) and radiological progression led to the introduction of everolimus and exemestane, without any significant response after nine months. Chemotherapy was started (six cycles of weekly paclitaxel; 3 weeks/4) with a decrease in CA15.3 and in bone FDG uptake. Three months after the last paclitaxel administration, based on the progression of bone metastases on PET-CT and re-increase in CA15.3 (350kU/L), capecitabine was started for eight cycles (14 days/21) and was interrupted due to asthenia, diarrhea, and progressive grade 2 anemia. In March 2021, an increase in CA15.3 (500kU/L) and progression on PET-CT led to the initiation of eribulin; however, this treatment was rapidly stopped after 3 months as CA15.3 continued to increase (900kU/L) and as PET-CT showed significant progression in size, number, and FDG metabolism of bone lesions. There was no FDG avid extra-skeletal metastasis, particularly nodal disease. At this time, complete blood count showed a grade 1 anemia (10.7g/dl), Lactate Dehydrogenase (LDH), and Phosphatase alkaline levels were 264U/L (normal range <225U/L) and 101 U/L (<130U/L), respectively. The patient had moderate and diffuse dorso-lumbar pain (Visual Analogue Scale 3/10). A ^99^mTc-Hydroxyethylene diphosphonate scintigraphy (^99^mTc-HDP bone scan) showed increased radiotracer uptake in multiple bone lesions of the entire axial skeleton, suggesting osteoblastic lesions. We started radium-223 treatment (off-label clinical use) in August 2021 in association with letrozole and denosumab. Radium-223 was administered at the same dose as for prostate cancer (50 kBq/kg every 4 weeks for six cycles) with a repeated complete blood count before each administration. Clinically, pain disappeared after two cycles of radium-223. A thoraco-abdominal CT after three cycles confirmed the absence of any new bone (or visceral) metastases and radium-223 was continued for two cycles and was well tolerated. The sixth administration was not done due to asymptomatic low hemoglobin (8.7 g/dl). The FDG PET-CT performed one month after the fifth cycle showed the development of three liver metastases (19mm, 16mm, and 10mm) and right axillary adenopathy (14mm), reflecting an extra-skeletal progression. However, a decrease in FDG uptake was observed in all bone metastatic lesions (**Figure 1**) that were previously described; for example, we observed a metabolic decrease of 59% in the 10th dorsal vertebra (with maximum standardized uptake value (max. SUV) of 12.4 before and 5.1 after radium-223) and of 63% in the sacrum (with max. SUV of 14.9 before and 5.4 after radium-223). The SUVmax in normal liver (reference) was 2.14 in pre- and 2.20 in post-radium-223 (+2.8%). Interestingly, the FDG uptake was increased in bilateral humerus, clavicules, and femur diaphysis, but this increased metabolism was homogenous and symmetrical, reflecting medullary regeneration. The ^99^mTc-HDP bone scan showed a decrease in ^99^Tc uptake in previously described bone lesions. There was a discrepancy between FDG metabolism decrease and ^99^Tc uptake increase in the humeral head (**Figure 2**), which likely reflects reparative bone associated with treatment response. Bone CT of the column showed hyperdensities of bone metastases, reflecting increased bone formation on treatment (**Figure 3**). CA15.3 level increased from 941 kU/L at baseline to 1135 kU/L after radium-223 which is attributed to the progressive liver and nodal metastases. Alkaline phosphatase and LDH remained in the normal range during radium-223 treatment. Cisplatin (70mg/m^2^) plus gemcitabine (1000mg/m^2^) was started 5 weeks after the last administration of radium-223, with G-CSF support. Three cycles were administered with a CA 15.3 response (decrease to 900 kU/L) and a grade 2 thrombopenia that resolved spontaneously. The timeline is summarized in **Figure 4**.

## Discussion

In this case report, we describe the treatment effect of radium-223 on bone metastasis in a patient with heavily pretreated and chemotherapy-refractory metastatic breast cancer. This case demonstrates the feasibility of this approach, even in a patient who had previously received multiple lines of systemic therapy. Radium-223 was very well tolerated and our patient remained free of symptoms during the treatment period. Asymptomatic grade 2 anemia (Common Terminology Criteria for Adverse Events) occurred after cycles but resolved spontaneously. Subsequent cisplatin-based chemotherapy was started after radium-223 treatment without any significant hematotoxicity.

Radium-223 appeared efficient in delaying bone progression. First, we observe a decrease in FDG uptake in all previously described bone metastases; ^99^mTc-HDP bone scan showed a decrease in uptake of bone lesions and an increase in bone densities on CT which indicate treatment response. Our findings suggest that FDG PET can be used to monitor radium-223 therapy. Second, the extra-skeletal disease progression in the liver and axillary adenopathy is in contrast with the disease control of bone lesions achieved with radium-223. This indicates the efficacy of radium-223 in treating bone metastasis despite the presence of extra-skeletal disease progression. Third, our patient did not experience an increase in pain or skeletal complication during treatment, despite a high baseline tumor burden in the skeletal. In a phase IIa nonrandomized study, Coleman et al. described the decrease of urinary N-telopeptide of type 1 and alkaline phosphatase after four cycles of radium-223 as potential biomarkers that reflect a positive treatment effect (5).

While our patient rapidly progressed during the previous chemotherapy regimen, the progression-free survival on radium-223 reached 6 months with an excellent quality of life, suggesting that radium-223 could represent an additional therapeutic option in metastatic breast cancer. Our patient with metastatic breast cancer was a good candidate for this treatment as the metastatic disease remained localized in the bone compartment with a follow-up of 6 years, suggesting a specific bone tropism in this case. The development of visceral lesions was expected in this patient, as part of the natural evolution of the cancer and the absence of an efficient systemic agent that was administered concomitantly; letrozole was deemed as the best choice but not an optimal choice as she previously progressed on different endocrine therapies. The evolution of liver metastasis was already described by Ueno et al.; 36 patients with metastatic breast cancer and bone-dominant disease received radium-223 with endocrine therapy. The 9-month disease control rate was 49% with a median progression-free survival of 7.4 months. Liver progression was observed in around 56% of patients (6). This emphasizes the need for evaluating the role of radium-223 earlier in the disease course and in combination with systemic agents. Different trials are currently evaluating the efficacy of radium-223 in breast cancer in combination with paclitaxel (NCT04090398), endocrine treatments (aromatase inhibitors plus everolimus; NCT02258451), and capecitabine (ISRCTN92755158) (8).

In conclusion, radium-223 could be a promising therapeutic option in well-selected patients with metastatic breast cancer with bone-only disease. However, careful consideration of hematologic adverse events is necessary for an optimal patient selection. Clinical trials with radium-223, particularly in combination with other agents, are ongoing to further improve clinical outcomes in patients with advanced breast cancer.

## Patient Perspective

Progression of cancer always induces fear of limitation in therapeutic options. With this treatment, I had a good quality of life and control of bone disease. The radium-223 therapy may be a great therapeutic option to existing treatments.

## Data Availability Statement

The raw data supporting the conclusions of this article will be made available by the authors, without undue reservation.

## Ethics Statement

Written informed consent was obtained from the patient for the publication of any potentially identifiable images or data included in this article.

## Author Contributions

HH followed the patient, evaluated response and wrote the manuscript. FD followed the patient, evaluated response and wrote the manuscript. DF followed the treatment, evaluated response and administered the treatment. ES followed the patient, evaluated response and wrote the manuscript. All authors contributed to the article and approved submitted version.

## Conflict of Interest

The authors declare that the research was conducted in the absence of any commercial or financial relationships that could be construed as a potential conflict of interest.

## Publisher’s Note

All claims expressed in this article are solely those of the authors and do not necessarily represent those of their affiliated organizations, or those of the publisher, the editors and the reviewers. Any product that may be evaluated in this article, or claim that may be made by its manufacturer, is not guaranteed or endorsed by the publisher.
